# Crystallization of Nano-Sized Macromolecules by the Example of Hexakis-[4-{(N-Allylimino)methyl}phenoxy]cyclotriphosphazene

**DOI:** 10.3390/nano12132268

**Published:** 2022-06-30

**Authors:** Evgeniy Chistyakov, Pavel Yudaev, Yulia Nelyubina

**Affiliations:** 1 Department of Chemical Technology of Polymers, Mendeleev University of Chemical Technology of Russia, Miusskaya Sq., 9, 125047 Moscow, Russia; yudaevpavel5@gmail.com; 2 Center for Molecular Composition Studies, A.N. Nesmeyanov Institute of Organoelement Compounds of Russian Academy of Sciences (INEOS RAS), 28 Vavilov Str., 119334 Moscow, Russia; unelya@ineos.ac.ru

**Keywords:** single crystal, crystal structure, nanoparticle, phosphazene, X-ray diffraction

## Abstract

The synthesized compound was characterized by ^31^P, ^13^C, and ^1^H NMR spectroscopy and MALDI-TOF mass spectroscopy. According to DSC data, the compound was initially crystalline, but the crystal structure was defective. The crystals suitable for X-ray diffraction study were prepared by slow precipitation of the compound from a solution by a vapor of another solvent. A study of the single crystal obtained in this way demonstrated that the phosphazene ring has a flattened chair conformation. It was found that the sphere circumscribed around the compound molecule has a diameter of 2.382 nm.

## 1. Introduction

Azomethines (Schiff bases) are promising compounds for use in various fields of science and technology: extraction of metals [1,2], analytical chemistry [3], chemosensorics [4], polymer photostabilization [5], etc. Azomethines inhibit steel corrosion in hydrochloric [6] and sulfuric [7] acid solutions since they are easily adsorbed at the metal/solution interface. Azomethines with various substituents at nitrogen and carbon atoms are applied in medicine and pharmacology owing to their antimicrobial action, lack of toxicity [8,9], and biological activity [10,11]. Schiff bases with benzothiazole and sulfonate groups inhibit a pancreatic enzyme: lipase; this is important for the treatment of obesity [12]. Polymer hydrogels containing azomethine bonds serve as drug carriers, as they undergo biodegradation via hydrolysis of the CH=N bond [13]. Chitosan linked to a polymeric Schiff base selectively extracts Au(III) and Ag(I) from acidic aqueous solutions (pH = 1) in the presence of divalent heavy metals: copper, lead, cadmium, and zinc [14].

Azomethines are precursors for the synthesis of new compounds with valuable properties. For example, O-phosphorylated derivatives of pyridoxal, a vitamin involved in biochemical processes, were prepared using azomethines [15].

Schiff bases can coordinate transition metals; therefore, they are used to manufacture palladium catalysts for the Suzuki–Miyaura cross-coupling [16] and Mizoroki–Heck reaction [17] and heteronuclear complexes with lanthanides [18], octahedral nickel(II) complexes [19], copper(II) [20,21,22,23], scandium(III) [24], and cobalt(II) [25] complexes, as well as redox-active complexes with germanium(IV) [26]. Palladium(II) and chromium(III) complexes formed by azomethines containing heterocycles inhibit the growth of *S. marcescent*, *E. coli*, and *M. luteus* bacteria and possess a pronounced antitumor activity against MCF-7, HepG-2, and HCT-116 cancer cells [27]. Nickel(II) and platinum(II) complexes with bis-azomethine, 2-(5-(*tert*-butyl)-2-hydroxybenzylidene)-*N*-cyclohexylhydrazine-1-carbothioamide, exhibit a considerable antibacterial activity against *E. coli* and *S. aureus* [28]. Azomethine metal complexes are used to manufacture additives for lubricating compositions [29]. Zinc(II) azomethine complexes exhibit photoluminescent properties and fungicidal activity [30].

Changing the molecular geometry of azomethines may afford liquid crystals with optical properties [31] and smectic crystalline materials for manufacturing organic field-effect transistors [32].

Azomethines and their metal complexes possess luminescent properties. Shienok et al. [33] obtained azomethine with tetraarylimidazole moieties possessing tunable luminescence for molecular switches. Azomethines containing benzodifuran-thiophene moieties are used to fabricate optoelectronic devices [34]. Azomethines based on tris(2-aminoethyl)amine show emission in the 380–515 nm range in the solid-state and are promising as components of light-emitting diodes [35]. Azomethines with pyrazole groups show a large Stokes shift in tetrachloromethane, which allows them to be used in fluorescent molecular probes [36]. Zinc complexes of azomethine ligands, *N,N*-bis(salicylaldehyde)-2,3-diaminonaphthalene and *N,N*-bis(1-naphthalidimine)-2,3-diaminonaphthalene, act as light-emitting components in the development of fluorescent sensors and organic display devices [37].

The introduction of azomethine bonds into aminolysed polyethylene terephthalate is a new method for processing hazardous polymer wastes in order to obtain metal chelates [38].

Aryloxycyclophosphazenes containing azomethine groups are of particular research interest. They are widely used in biomedicine [39], as flame retardant materials [40], for coordination chemistry and catalysis [41], and as fluorescent dyes [42]. This is due to their polyfunctionality, biocompatibility, high thermal stability, flame resistance, and UV radiation resistance. Aslan F. et al. [43,44] synthesized several dozens of aryloxycyclophosphazenes with various substituents at the azomethine group formed by hexakis-[(4-formyl)phenoxy]cyclotriphosphazene and hexakis-[(2-formyl)phenoxy]cyclotriphosphazene. However, nothing was known about the crystal structure of azomethine derivatives. The main challenge was to obtain a single crystal. The single crystals of low-molecular-weight azomethines are often obtained by evaporation of the solvent from, for example, ethanol solutions [8]. In the case of the high-molecular-weight compounds of a complex architecture, most of the existing methods are inapplicable. Therefore, in this study, we tested a new method using specially synthesized hexakis-[4-{(N-allylimino)methyl}phenoxy]cyclotriphosphazene. Finally, we obtained a single crystal of this compound and studied it by X-ray diffraction.

## 2. Materials and Methods

### 2.1. Materials

Hexachlorocyclotriphosphazene, 99% (Fushimi Pharmaceutical Co., Ltd., Marugame, Kagawa Prefecture, Japan). 4-Hydroxybenzaldehyde, 98%; allylamine, 98%; magnesium sulfate, anhydrous, ≥99.5%; dichloromethane, ≥99.5%; chloroform, anhydrous, ≥99%; tetrahydrofuran, anhydrous, ≥99.9%; sodium, 99.9%; ethanol, anhydrous, ≥99.5%; toluene, anhydrous, 99.8%; hexane, anhydrous, 95% (Sigma-Aldrich, Saint Louis, MO, USA).

### 2.2. Methods

^1^H, ^13^C, and ^31^P NMR spectra were recorded on an Agilent/Varian Inova 400 spectrometer (Agilent Technologies, Santa Clara, CA, USA) operating at 400.02 MHz, 100.60 MHz, and 161.94 MHz, respectively. MALDI-TOF mass spectrometry data were acquired using a Bruker Auto Flex II mass spectrometer (Bruker, Billerica, MA, USA). Differential scanning calorimetry (DSC) measurements were done using a NETZSCH STA 449F1 instrument (Erich NETZSCH GmbH & Co. Holding KG, Selb, Germany).

Crystallographic data: Crystals of APP (C_60_H_60_N_9_O_6_P_3_, M = 1096.08) are monoclinic, space group P2_1_/n; at 296 K: a = 8.4373(2), b = 14.9917(3), c = 46.2319(8) Å, β = 94.4940(10)°, V = 5829.9(2) Å^3^, Z = 4, d_calc_ = 1.249 g cm^−3^, μ(MoKα) = 1.60 cm^−1^, and F(000) = 2304. The intensities of 66102 reflections were measured with a Bruker Quest D8 CMOS diffractometer (Bruker AXS, Madison, USA) [λ(MoKα) = 0.71073 Å, ω-scans, 2θ < 54°], and 12686 independent reflections were used for further refinement. Using Olex2 [45], the structure was solved with the ShelXT [46] structure solution program using Intrinsic Phasing and refined with the XL [46] refinement package using least-squares minimization. Hydrogen atom positions were calculated and refined in the isotropic approximation within the riding model. The refinement converged to wR2 = 0.2024 and GOF = 1.097 for all the independent reflections (R1 = 0.0782 was calculated against F for 8108 observed reflections with I > 2σ(I)). CCDC 2174092 contains the supplementary crystallographic; information for this paper.

The size of the APP molecule was determined by processing X-ray diffraction data using Mercury 3.8 software (created by CCDC, CSD license).

### 2.3. Synthesis of Hexakis-[(4-formyl)phenoxy]cyclotriphosphazene (FPP)

Hexakis-[4-formylphenoxy]cyclotriphosphazene was synthesized by the procedure described in a previous study [47].

4-Hydroxybenzaldehyde (7.32 g, 0.06 mol) was dissolved in ethanol (30 mL) in a three-necked flask equipped with a stirrer and a reflux condenser. After complete dissolution of 4-hydroxybenzaldehyde, the alcohol solution of sodium ethylate, which was obtained via dissolution of sodium (1.15 g, 0.05 mol) in ethanol (20 mL), was loaded into the flask. The reaction time was 10 min. Then, ethanol was distilled off on a rotary evaporator in vacuum. The residue was dried in vacuum up to a constant weight. The yield of the product was quantitative.

4-Hydroxybenzaldehyde phenolate (8.64 g, 0.06 mol) was loaded in a three-necked flask equipped with a stirrer and a reflux condenser, and tetrahydrofuran (40 mL) was added. A solution of hexachlorocyclotriphosphazene (2.61 g, 0.0075 mol) in tetrahydrofuran (20 mL) was added to the dispersion formed during stirring. The time of reaction was 9 h during solvent boiling. When the process was complete, the reaction mixture was filtered off and the mother liquor was evaporated on a rotary evaporator. The product was recrystallized from the ethanol–chloroform mixture. Yield: 4.52 g (70%).

### 2.4. Synthesis of Hexakis-[4-{(N-allylimino)methyl}phenoxy]cyclotriphosphazene (APP)

Hexakis-[(4-formyl)phenoxy]cyclotriphosphazene (0.5 g, 0.5807 mmol) was charged into a 50 mL round-bottom flask and dissolved in dichloromethane (5 mL). After complete dissolution, allylamine (0.31 mL, 4.181 mmol) and magnesium sulfate (0.56 g, 4.646 mmol) were added. The reaction mixture was magnetically stirred for 48 h at 25 °C. The solution was decanted from magnesium sulfate, and dichloromethane was evaporated on a rotary evaporator. The product was dried in a drying oven under vacuum at 40 °C for 4 h. Yield: 0.57 g (90%). The product was recrystallized from a toluene–hexane solvent system.

## 3. Results and Discussion

**Figure 1:**
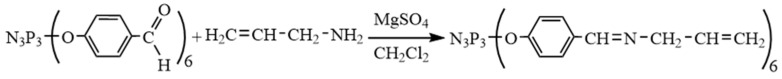
Synthesis of APP.

The synthesis of APP was performed according to the scheme shown in Figure 1. The reaction was conducted in a non-polar solvent to facilitate the removal of water. Magnesium sulfate was used as the drying agent. A 20% molar excess of allylamine over aldehyde groups was used to ensure complete aldehyde to azomethine conversion.

The excess allylamine was removed under vacuum at a temperature not higher than 40 °C, together with the solvent. In this case, the residue was a loose crystalline material. At higher temperature, an amorphous glassy material was formed, which later turned yellow as a result of side reactions.

**Figure 2:**
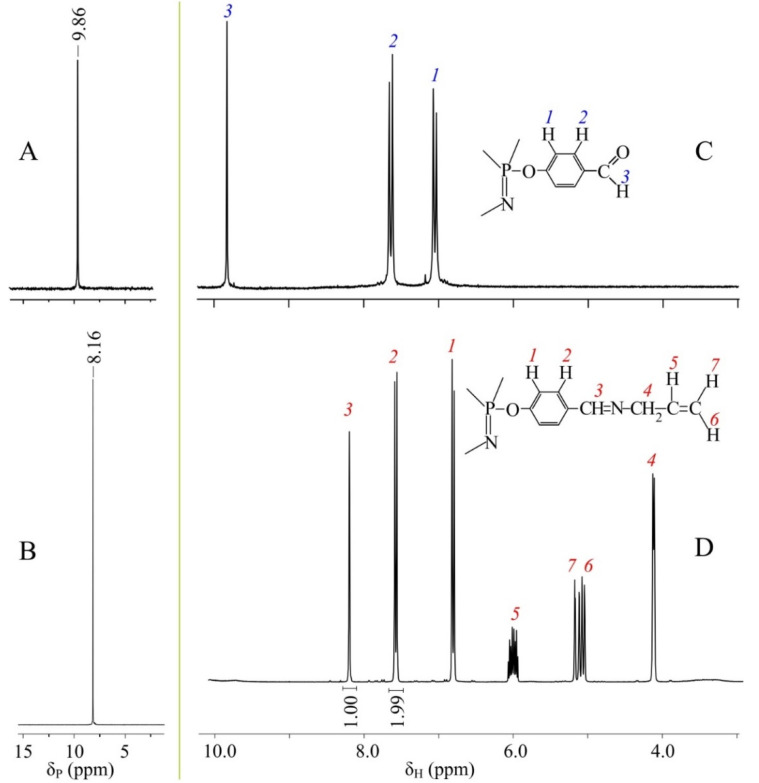
^31^P NMR spectra FPP (**A**) and APP (**B**) and ^1^H NMR spectra of FPP (**C**) and APP (**D**).

The phosphorus NMR spectrum of the product is a singlet, indicating the absence of side reactions affecting the phosphazene ring (Figure 2B). This singlet is shifted by 1.7 ppm relative to the signal of the initial FPP (Figure 2A). This is caused by the difference between mesomeric effects of the aldehyde and azomethine groups affecting the phosphorous atoms of the phosphazene ring.

A similar picture is observed for the ^1^H NMR spectrum of the product (Figure 2D). The azomethine proton signal is shifted upfield by approximately 1.7 ppm relative to the proton signal of the aldehyde group (Figure 2C). The absence of a signal at 9.8 ppm in the spectrum of APP means that the aldehyde groups were completely converted to azomethine groups.

**Figure 3:**
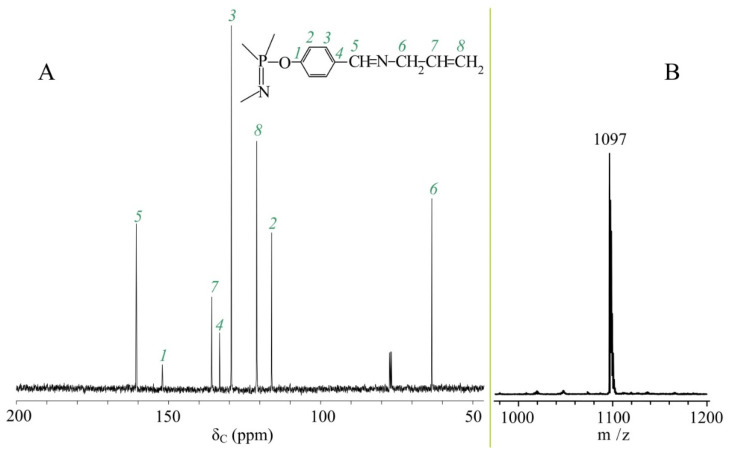
^13^C NMR spectra (**A**) and MALDI-TOF mass spectra (**B**) of APP.

The completeness of the conversion is confirmed by ^13^C NMR spectra (Figure 3A) and MALDI-TOF mass spectra (Figure 3B).

The ^13^C NMR spectrum shows a carbon signal for the azomethine group at 161 ppm, but no signal typical of the aldehyde group carbon at 191 ppm.

The MALDI-TOF mass spectrum has only one peak corresponding to the mass of the target product solvated by the matrix proton (1096 + H^+^).

**Figure 4:**
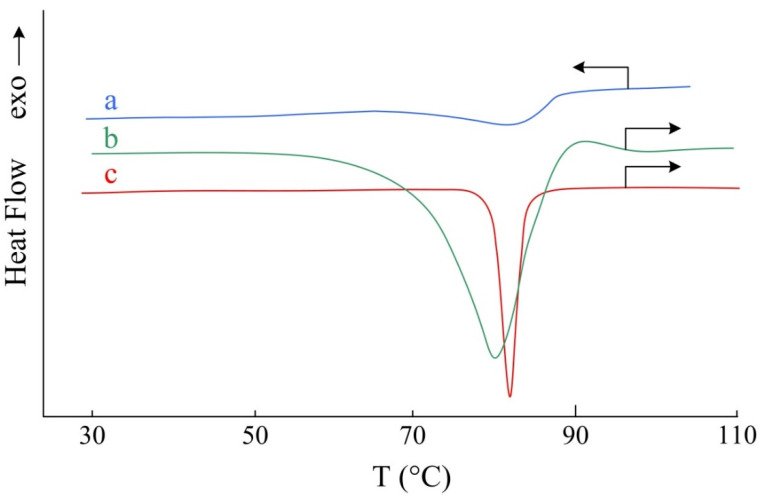
DSC curves of APP: heating before recrystallization (**b**) and after recrystallization (**c**) and cooling (**a**).

According to the DSC study of APP, the product was crystalline, but melted in a wide temperature range from 50 to 90 °C (Figure 4b). This attests to a polycrystalline structure with numerous defects.

**Figure 5:**
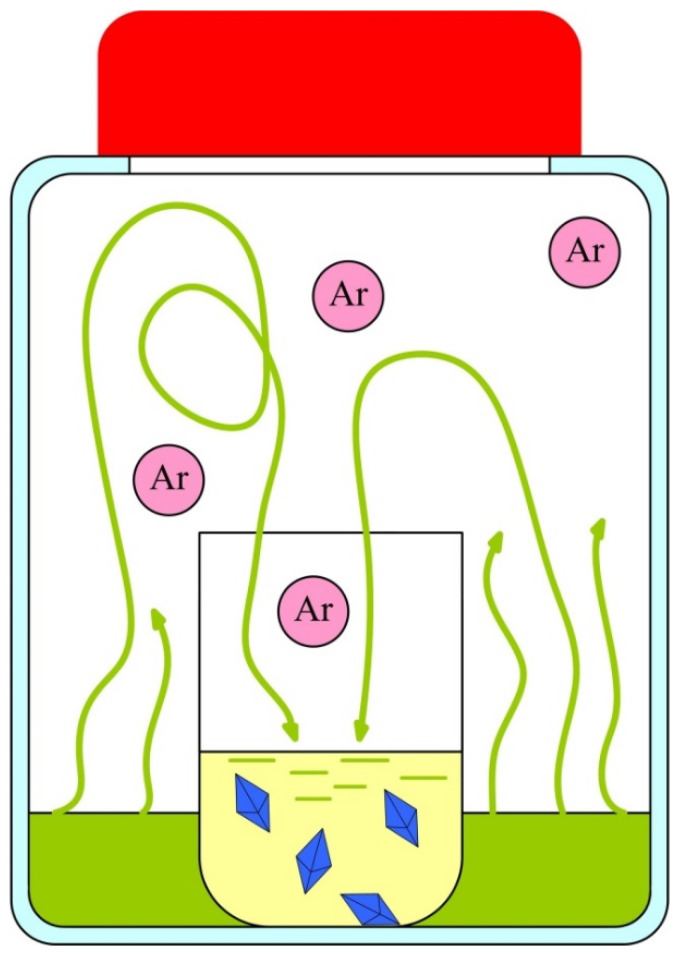
Crystallization in a confined space.

Considering the cooling curve shows that APP does not crystallize from the melt, since only a heat capacity step corresponding to the glass transition of the sample is observed. Hence, methods for the preparation of single crystals involving sample heating are inapplicable in this case. Methods of low-temperature evaporation of the solvent were also tested; however, with volatile solvents, crystallization took place rapidly and a single crystal was not formed. In the case of low-volatile solvents, crystallization was slow, but was accompanied by oxidation and yellowing of the compound. Therefore, we proposed an alternative method for the preparation of single crystals, which was based on the precipitation of the compound by the vapor of a volatile solvent from a solution in a low-volatile solvent in a confined space. The vessel in which crystallization took place was filled with an inert gas (argon). The method is based on the use of a closed system in which two liquids with different volatility are placed, separated by a partition. Accordingly, the filling of the volume with vapors of these liquids begins, while the volume is saturated with vapors of a more volatile liquid. Since the pressure of saturated vapor over a slightly volatile liquid is less than over a highly volatile liquid, slow diffusion of the vapors of a volatile liquid into a slightly volatile liquid occurs. If, however, a solution of a crystalline substance is prepared in a slightly volatile liquid and a highly volatile liquid is used in which the substance does not dissolve, the substance will be gradually displaced by vapors of the volatile liquid from the solution and form crystals (Figure 5).

In our case, toluene was chosen as a slightly volatile liquid in which a solution of APP was prepared. Hexane was chosen for precipitation as it is inert, readily dehydrated, and can dissolve the residual allylamine and dichloromethane. Deposition with hexane vapor led to the formation of relatively large hexagonal lamellar single crystals with an average face size of about 1 mm, suitable for X-ray diffraction studies.

The DSC curve of the recrystallized and dried APP sample shows a melting peak in a narrow temperature range (Figure 4c) of 78–85 °C with a maximum at 82 °C, which indirectly confirms the ordering of the sample structure and the absence of defects.

**Figure 6:**
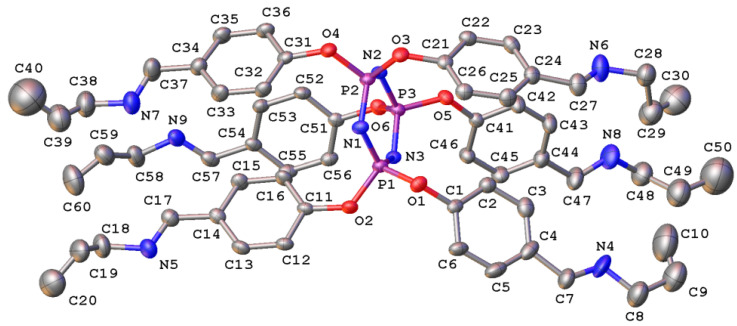
General view of the compound APP in the representation of non-hydrogen atoms as thermal ellipsoids at a 20% probability level. Hydrogen atoms as well as minor components of the disordered allyl groups are omitted for clarity.

The single crystals grown by the developed method were studied by X-ray diffraction (Figure 6). The X-ray diffraction data from a single crystal were collected at room temperature and accessed using no operational low-temperature device. At this temperature, the heterocyclic P_3_N_3_ core adopts a flattened chair conformation, with the atoms P(2) and N(3) deviating from the mean plane of the other atoms by 0.256(3) and 0.156(3) Å, respectively. The substituents reside at the phosphorus atoms in the axial positions on both sides of the P_3_N_3_ core. The phenyl rings of the neighboring substituents are rotated so that no intramolecular stacking interactions occur between them; the angles of the two such rings relative to the third one on the opposite sides of the P_3_N_3_ core are 29.25(13), 53.91(15) and 10.21(13), 56.88(13)°, respectively. The lack of intermolecular stacking interactions may arise from the bulky allyl groups that are severely disordered at the accessed temperature and thereby prevent the phenyl groups from approaching each other. As a result, the molecules of APP are held together only by weak van der Waals contacts. More detailed information about the APP structure is presented in the Supplementary Materials.

It was shown that the sphere circumscribed around the APP macromolecule has a diameter of 2.382 nm, which allows assigning the molecule to nano-sized structures.

## 4. Conclusions

Thus, it can be concluded that the developed method for crystallization of macromolecules is applicable for the preparation of single crystals of high-molecular-weight (1096 Da) compounds with complex architecture. These macromolecules fall into the nanoscale range and can be used as nanoparticles in various fields of science and technology, for example, as modifying agents for polymer materials and as drug carriers, catalysts, chelators, and so on.

## Supplementary Materials

The following supporting information can be downloaded at: https://www.mdpi.com/article/10.3390/nano12132268/s1, the files APP.cif and checkcif_APP.pdf with information about the APP structure are presented.
