# Supplementary material for: Crystallization of Nano-Sized Macromolecules by the Example of Hexakis-[4-{(N-Allylimino)methyl}phenoxy]cyclotriphosphazene
Source: Nanomaterials (Basel). 2022 Jun 30;12(13):2268. doi: 10.3390/nano12132268 (PMC9268015; doi:10.3390/nano12132268)

checkCIF/PLATON report

Structure factors have been supplied for datablock(s) APP

THIS REPORT IS FOR GUIDANCE ONLY. IF USED AS PART OF A REVIEW PROCEDURE FOR PUBLICATION, IT SHOULD NOT REPLACE THE EXPERTISE OF AN EXPERIENCED CRYSTALLOGRAPHIC REFEREE.

No syntax errors found. CIF dictionary Interpreting this report

Datablock: APP

```
Bond precision:   C-C = 0.0056 A          Wavelength=0.71073

Cell:              a=8.4373(2)     b=14.9917(3)        c=46.2319(8)
                   alpha=90        beta=94.494(1)      gamma=90
Temperature:       296 K

                 Calculated                         Reported
Volume           5829.9(2)                          5829.9(2)
Space group      P 21/n                             P 1 21/n 1
Hall group       -P 2yn                             -P 2yn
Moiety formula   C60 H60 N9 O6 P3                   C60 H60 N9 O6 P3
Sum formula      C60 H60 N9 O6 P3                   C60 H60 N9 O6 P3
Mr               1096.08                            1096.08
Dx,g cm-3        1.249                              1.249
Z                4                                  4
Mu (mm-1)        0.160                              0.160
F000             2304.0                             2304.0
F000'            2306.07
h,k,lmax         10,19,59                           10,19,59
Nref             12710                              12686
Tmin,Tmax        0.972,0.984                        0.591,0.746
Tmin'            0.969

Correction method= # Reported T Limits: Tmin=0.591 Tmax=0.746
AbsCorr = MULTI-SCAN

Data completeness= 0.998            Theta(max)= 26.999

                                                       wR2(reflections)=
R(reflections)= 0.0782( 8108)                          0.2024( 12686)

S = 1.075                    Npar= 769
```

The following ALERTS were generated. Each ALERT has the format
test-name_ALERT_alert-type_alert-level.
Click on the hyperlinks for more details of the test.

Alert level B

PLAT220_ALERT_2_B NonSolvent Resd 1 C Ueq(max)/Ueq(min) Range 7.2 Ratio

Author Response: All allyl groups are severely disordered at the accessed temperature.

PLAT234_ALERT_4_B Large Hirshfeld Difference C29 --C30 . 0.27 Ang.

Author Response: All allyl groups are severely disordered at the accessed temperature.

Alert level C

PLAT220_ALERT_2_C NonSolvent Resd 1 N Ueq(max)/Ueq(min) Range 3.1 Ratio

Author Response: All allyl groups are severely disordered at the accessed temperature.

PLAT222_ALERT_3_C NonSolvent Resd 1 H Uiso(max)/Uiso(min) Range 6.3 Ratio
PLAT234_ALERT_4_C Large Hirshfeld Difference N4 --C7 . 0.24 Ang.

Author Response: All allyl groups are severely disordered at the accessed temperature.

PLAT234_ALERT_4_C Large Hirshfeld Difference N4 --C8 . 0.23 Ang.

Author Response: All allyl groups are severely disordered at the accessed temperature.

PLAT234_ALERT_4_C Large Hirshfeld Difference N8 --C48 . 0.20 Ang.

Author Response: All allyl groups are severely disordered at the accessed temperature.

PLAT234_ALERT_4_C Large Hirshfeld Difference C8 --C9 . 0.22 Ang.

Author Response: All allyl groups are severely disordered at the accessed temperature.

PLAT234_ALERT_4_C Large Hirshfeld Difference C19 --C20 . 0.23 Ang.

Author Response: All allyl groups are severely disordered at the accessed temperature.

```
PLAT234_ALERT_4_C Large Hirshfeld Difference C48      --C49      .       0.17 Ang.
```

Author Response: All allyl groups are severely disordered at the accessed temperature.

```
PLAT241_ALERT_2_C High   'MainMol' Ueq as Compared to Neighbors of         C5 Check
PLAT242_ALERT_2_C Low    'MainMol' Ueq as Compared to Neighbors of        C44 Check
PLAT260_ALERT_2_C Large Average Ueq of Residue Including       P1      0.114 Check
PLAT334_ALERT_2_C Small <C-C> Benzene Dist.   C1       -C6       .       1.37 Ang.
PLAT334_ALERT_2_C Small <C-C> Benzene Dist.   C21      -C26      .       1.37 Ang.
PLAT334_ALERT_2_C Small <C-C> Benzene Dist.   C31      -C36      .       1.37 Ang.
PLAT334_ALERT_2_C Small <C-C> Benzene Dist.   C41      -C46      .       1.37 Ang.
PLAT340_ALERT_3_C Low Bond Precision on  C-C Bonds ..............    0.00559 Ang.
PLAT906_ALERT_3_C Large K Value in the Analysis of Variance ......     24.132 Check
PLAT906_ALERT_3_C Large K Value in the Analysis of Variance ......      4.064 Check
PLAT906_ALERT_3_C Large K Value in the Analysis of Variance ......      2.032 Check
PLAT911_ALERT_3_C Missing FCF Refl Between Thmin & STh/L=    0.600          7 Report
```

Alert level G

```
PLAT002_ALERT_2_G Number of Distance or Angle Restraints on AtSite         33 Note
PLAT003_ALERT_2_G Number of Uiso or Uij Restrained non-H Atoms ...         28 Report
PLAT171_ALERT_4_G The CIF-Embedded .res File Contains EADP Records         20 Report
PLAT172_ALERT_4_G The CIF-Embedded .res File Contains DFIX Records         18 Report
PLAT176_ALERT_4_G The CIF-Embedded .res File Contains SADI Records         10 Report
PLAT186_ALERT_4_G The CIF-Embedded .res File Contains ISOR Records         18 Report
PLAT230_ALERT_2_G Hirshfeld Test Diff for    C9       --C10      .        7.5 s.u.
PLAT230_ALERT_2_G Hirshfeld Test Diff for    C39      --C40      .        8.0 s.u.
PLAT230_ALERT_2_G Hirshfeld Test Diff for    C49      --C50      .        6.5 s.u.
PLAT301_ALERT_3_G Main Residue  Disorder .............(Resd  1  )        26% Note
PLAT410_ALERT_2_G Short Intra H...H Contact  H17      ..H18A     .       2.07 Ang.
                                                        x,y,z  =     1_555 Check
PLAT410_ALERT_2_G Short Intra H...H Contact  H27      ..H28D     .       2.09 Ang.
                                                        x,y,z  =     1_555 Check
PLAT410_ALERT_2_G Short Intra H...H Contact  H57      ..H58A     .       2.09 Ang.
                                                        x,y,z  =     1_555 Check
PLAT410_ALERT_2_G Short Intra H...H Contact  H57      ..H58D     .       2.03 Ang.
                                                        x,y,z  =     1_555 Check
PLAT720_ALERT_4_G Number of Unusual/Non-Standard Labels .........          2 Note
PLAT811_ALERT_5_G No ADDSYM Analysis: Too Many Excluded Atoms ....          ! Info
PLAT860_ALERT_3_G Number of Least-Squares Restraints ............        197 Note
PLAT910_ALERT_3_G Missing # of FCF Reflection(s) Below Theta(Min).          4 Note
PLAT912_ALERT_4_G Missing # of FCF Reflections Above STh/L=  0.600         14 Note
PLAT960_ALERT_3_G Number of Intensities with I  <   - 2*sig(I) ...          4 Check
PLAT967_ALERT_5_G Note: Two-Theta Cutoff Value in Embedded .res ..       54.0 Degree
PLAT978_ALERT_2_G Number C-C Bonds with Positive Residual Density.          1 Info
```

---

0 **ALERT level A** = Most likely a serious problem - resolve or explain
2 **ALERT level B** = A potentially serious problem, consider carefully
20 **ALERT level C** = Check. Ensure it is not caused by an omission or oversight
22 **ALERT level G** = General information/check it is not something unexpected

0 ALERT type 1 CIF construction/syntax error, inconsistent or missing data

```
19 ALERT type 2 Indicator that the structure model may be wrong or deficient
10 ALERT type 3 Indicator that the structure quality may be low
13 ALERT type 4 Improvement, methodology, query or suggestion
 2 ALERT type 5 Informative message, check
```

It is advisable to attempt to resolve as many as possible of the alerts in all categories. Often the minor alerts point to easily fixed oversights, errors and omissions in your CIF or refinement strategy, so attention to these fine details can be worthwhile. In order to resolve some of the more serious problems it may be necessary to carry out additional measurements or structure refinements. However, the purpose of your study may justify the reported deviations and the more serious of these should normally be commented upon in the discussion or experimental section of a paper or in the "special_details" fields of the CIF. checkCIF was carefully designed to identify outliers and unusual parameters, but every test has its limitations and alerts that are not important in a particular case may appear. Conversely, the absence of alerts does not guarantee there are no aspects of the results needing attention. It is up to the individual to critically assess their own results and, if necessary, seek expert advice.

Publication of your CIF in IUCr journals

A basic structural check has been run on your CIF. These basic checks will be run on all CIFs submitted for publication in IUCr journals (*Acta Crystallographica*, *Journal of Applied Crystallography*, *Journal of Synchrotron Radiation*); however, if you intend to submit to *Acta Crystallographica Section C* or *E* or *IUCrData*, you should make sure that full publication checks are run on the final version of your CIF prior to submission.

Publication of your CIF in other journals

Please refer to the *Notes for Authors* of the relevant journal for any special instructions relating to CIF submission.

PLATON version of 18/05/2022; check.def file version of 17/05/2022

Datablock APP - ellipsoid plot

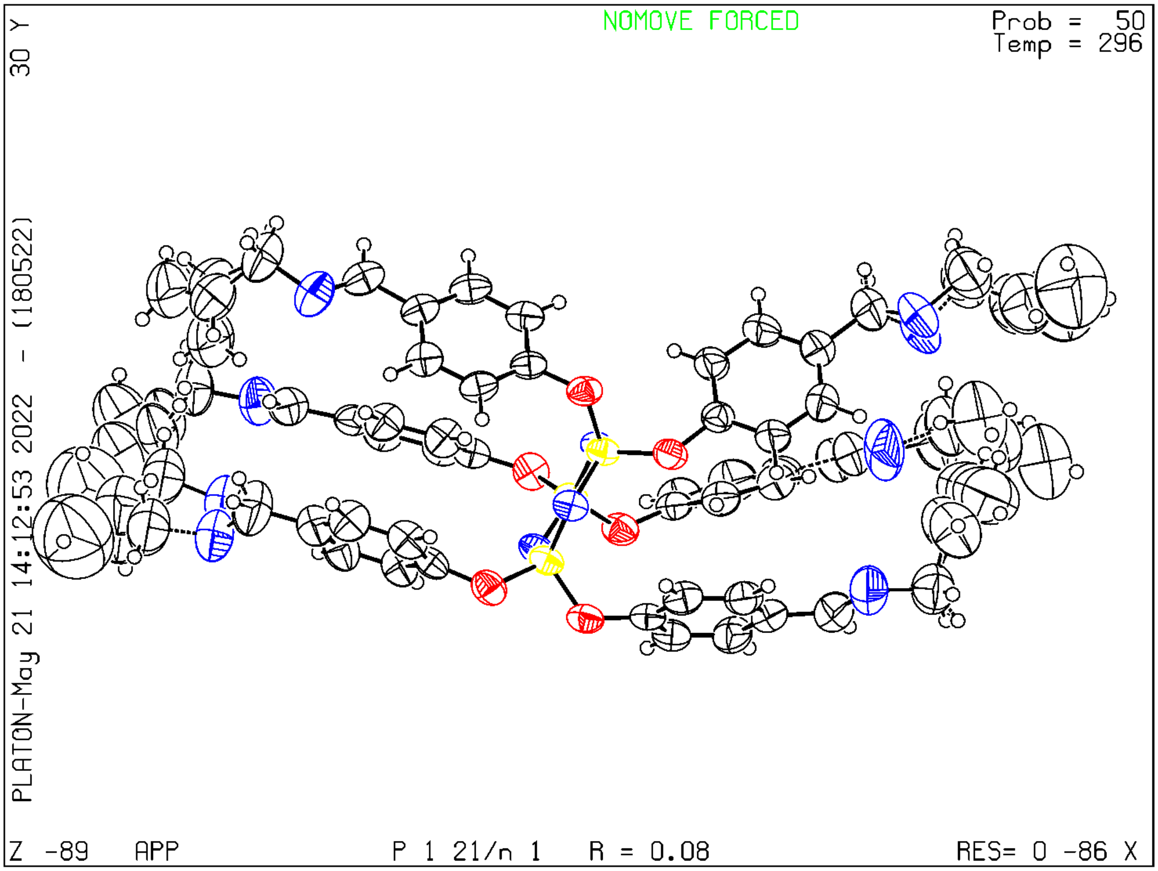

Supplement: Supplementary file 1 [file nanomaterials-12-02268-s001.zip › Supplementary Materials/checkcif_APP.pdf]
